# Additional capecitabine use in early-stage triple negative breast cancer patients receiving standard chemotherapy: a new era? A meta-analysis of randomized controlled trials

**DOI:** 10.1186/s12885-022-09326-5

**Published:** 2022-03-12

**Authors:** Feng Ye, Lei Bian, Jiahuai Wen, Ping Yu, Na Li, Xiaoming Xie, Xi Wang

**Affiliations:** 1 Department of Breast Oncology, Sun Yat-Sen University Cancer Center, State Key Laboratory of Oncology in South China, Collaborative Innovation Center for Cancer Medicine, 651 East Dongfeng Road, Guangzhou, Guangzhou, 510060 Guangdong China; 2 Department of Breast Oncology, Guangdong Hospital of Traditional Chinese Medicine, Guangzhou, Guangdong China; 3 Department of Anesthesiology, Sun Yat-Sen University Cancer Center, State Key Laboratory of Oncology in South China, Collaborative Innovation Center for Cancer Medicine, Guangzhou, Guangdong China

**Keywords:** Triple negative breast cancer, Standard chemotherapy, Additional capecitabine, Survival, Meta-analysis

## Abstract

**Background:**

The efficiency of capecitabine has been proven in early-stage triple negative breast cancer (eTNBC) with residue invasive tumor (non-pCR) after standard neoadjuvant chemotherapy (NACT). However, for those unselected eTNBC patients without screening from NACT (i.e., newly diagnosed eTNBC patients undergoing breast surgery followed by adjuvant systemic therapy), the value of capecitabine has still remains unclear. We performed a meta-analysis of randomized controlled trials (RCTs) to evaluate whether additional capecitabine in eTNBC patients could improve clinical outcomes.

**Methods:**

Seven RCTs (USO 01062, FinXX, GEICAM/2003, CREATE-X, CIBOMA/2004, CBCSG-010 and SYSUCC-001) were identified in online databases until December 2020 and included in the meta-analysis. We extracted the survival data including disease/relapse-free survival (DFS/RFS) and overall survival (OS), and utilized the STATA software to calculate the summarized hazard ratios (HRs) and 95% confidence intervals (95%CIs).

**Results:**

A total of 3329 eTNBC patients were enrolled in this meta-analysis, with 1640 receiving standard neo−/adjuvant chemo-regimes alone, and the other 1689 receiving an additional capecitabine use, respectively. Both DFS and OS were significantly improved with the addition of capecitabine, and the benefits remained consistent in those unselected eTNBC patients without screening from NACT. Subgroup analysis further proved that this improvement in DFS was significant in both nodal negative and positive patients. Similar benefits are also found across menopausal status (both pre- and post-menopause). Regarding toxicity, the hand-foot syndrome and neutropenia are the most common capecitabine related adverse events, and are mostly tolerable.

**Conclusions:**

The present meta-analysis of RCTs demonstrates for the first time that adding capecitabine to standard chemo-regimens could improve both DFS and OS in unselected eTNBC patients, and this benefit remains consistent regardless of nodal status and menopausal status, which may lead eTNBC therapy into a new era.

## Background

Triple Negative Breast Cancer (TNBC), i.e., simultaneously negative for ER, PR and HER-2, which accounts for approximately 15–20% of breast cancer (BC), has significantly inferior clinical outcomes than non-TNBC [1, 2]. Chemotherapy is generally the dominant systemic treatment for TNBC. To date, anthracyclines(A/E) and taxanes(T) have been the most popularly used and efficient cytotoxic drugs, and the A&T based chemo-regimens, along with other chemotherapy drugs, such as cyclophosphamide (CTX/C) or carboplatin (Cb), have been recommended as the preferred or standard chemo-regimens for early-stage TNBC (eTNBC) [3]. Moreover, dose-dense regimens are more effective than conventional schemes from past decades [4–7]. Neoadjuvant chemotherapy (NACT) has also offered important guidance for ameliorating prognosis, especially for TNBC or HER-2+ patients [8–10]. However, even with all the progress above, the prevention of tumor recurrence is still unsatisfactory for eTNBC.

Capecitabine(X) is an orally available prodrug of fluorouracil (5-Fu, F). Capecitabine is efficient and is used as a second-line monotherapy option in metastatic BC patients resistant to anthracycline and taxane based regimens [11, 12]. In recent years, several studies have explored the effect of adding capecitabine to standard chemotherapy regimens on improving clinical outcomes for early-stage TNBC [13, 14]. The two common strategies are adding capecitabine use concurrently with or sequentially after anthracycline and/or taxane based chemo-regimens [15]. The CREATE-X trial demonstrated that disease-free survival (DFS) was significantly prolonged with adjuvant capecitabine use in HER2-BC patients (especially in TNBC patients) who had a residual invasive disease (non-pCR) after standard NACT [16]. Thus, the addition of adjuvant capecitabine for eTNBC with non-pCR after NACT has been recommended in most guidelines [3].

However, for those unselected eTNBC patients without screening from NACT (i.e., newly diagnosed eTNBC patients undergoing breast surgery followed by adjuvant systemic therapy), the effectiveness of adding capecitabine is not convincing in previous randomized control trials (RCTs). The GEICAM/2003–10 showed that 4 cycles of capecitabine following 4 cycles of ET regimens might be inferior to the standard 4EC-4 T regimen in node positive eTNBC subgroup [17]. In the CIBOMA/2004–01 trial, 8 cycles of capecitabine following standard chemotherapy failed to significantly prolong DFS of whole TNBC, although in the subgroup analysis, non-basal TNBC patients seemed to gain a survival benefit [18]. Due to these negative results, the indications of additional capecitabine use, for unselected eTNBC patients without screening from NACT, have not been defined yet [19]. Recently, two important RCTs reported encouraging results. In the SYSUCC-001 trial with 434 unselected eTNBC patients enrolled, 5-year DFS was significantly prolonged in patients receiving additional capecitabine after standard chemotherapy (82.8% vs. 73.0%, *p* = 0.03, [20]. Similarly, the CBCSG-010 trial, which added capecitabine concurrently with the standard chemotherapy (3XAC-3TX vs. 3FAC-3 T), also obtained a favourable outcome for the capecitabine group [21].

With increasing and conflicting results reported by different trials, we performed the present meta-analysis of RCTs to evaluate the role of additional capecitabine in eTNBC receiving standard chemotherapy, especially in unselected patients without screening from NACT. In this study, we compared the survival outcomes and performed stratified analysis to identify the patients who most warranted additional capecitabine.

## Methods

### Publication selection and search strategy

This investigation is complied with the guidelines of Preferred Reporting Items for Systematic Reviews and Meta-analyses (PRISMA) and is registered at PROSPERO 2021 (ID:CRD42021227868; Available from: https://www.crd.york.ac.uk/prospero/display_record.php?ID=CRD42021227868).

Literature published before December 20, 2020, was identified in PubMed, EMBASE, and the Cochrane Library independently by FY and LB. Presentations of important annual conferences within the past 15 years, including the ESMO (European Society for Medical Oncology), SABCS (San Antonio Breast Cancer Symposium), and ASCO (American Society of Clinical Oncology), are also searched.

The following key words were used as queries: (breast OR mammary) AND (cancer OR neoplasm OR carcinoma) AND (capecitabine OR xeloda) AND (TNBC OR triple-negative OR triple negative OR basal-like OR HER-2 negative). Only studies written in English were included.

### Inclusion and exclusion criteria

The criteria for the included prospective RCTs were as follows: (1) non-metastatic TNBC patients with stage I-III disease were enrolled, even as a subgroup; (2) studies should compare the survival between the Control group (TNBC patients receiving standard neo−/adjuvant anthracyclines and/or taxanes based chemotherapy) and the Capecitabine group (TNBC patients receiving additional capecitabine); (3) detailed survival data, such as rates or hazard ratios (HRs) of overall survival (OS), disease-free survival (DFS) or relapse free survival (RFS), should be provided. Definitions of survival data should be described in these studies; and (4) only the latest report with complete survival data of the same RCT was included.

The exclusion criteria for trials were as follows: (1) non-human studies or Non-RCTs; (2) single-arm trials or studies without proper control groups; (3) studies without detailed survival data for analysis; and (4) ongoing trials without published results.

### Data extraction

The following information was independently extracted from eligible studies by FY and LB, independently: authors, study name, publication year, type of study (with registry number), the sample size of TNBC patients in each arm, median follow-up, baseline situation of TNBCs (unselected patients or screened from NACT), detailed chemotherapy regimens, capecitabine dosage and usage, enrolled clinical stage, nodal and menopausal status, and rate of adverse effects (AEs) if available. HR and 95%CI for survival data were extracted if available, or transformed from the survival curve [22].

The primary research objective was to evaluate the survival benefits of additional capecitabine in eTNBC patients, and the major endpoints were DFS/RFS and OS. The secondary research objective was to evaluate the toxicities, and the corresponding indices were AEs.

### Statistical analysis

The detailed survival data were extracted and summarized as HRs and corresponding 95% confidence intervals (95%CIs). Heterogeneity of these data among the eligible studies was detected by Chi-square test-based Q statistics and *I*^*2*^ test. If significant heterogeneity was indicated (i.e., *p* < 0.05/*I*^*2*^ > 50%), we applied a random-effect model to calculate the summarized HRs and 95%CI. Subgroup analyses were conducted according to lymph node status, menopausal status and regimens with/without taxanes. The results are shown in forest plots.

Egger’s test (indicated by *p* < 0.05) and funnel plots were utilized to evaluate the publication bias. All statistical analyses were performed with Stata 12.0 software (Stata Corporation, College Station, TX, USA) with two-sided *p* values.

## Results

### Eligible studies

**Fig. 1:**
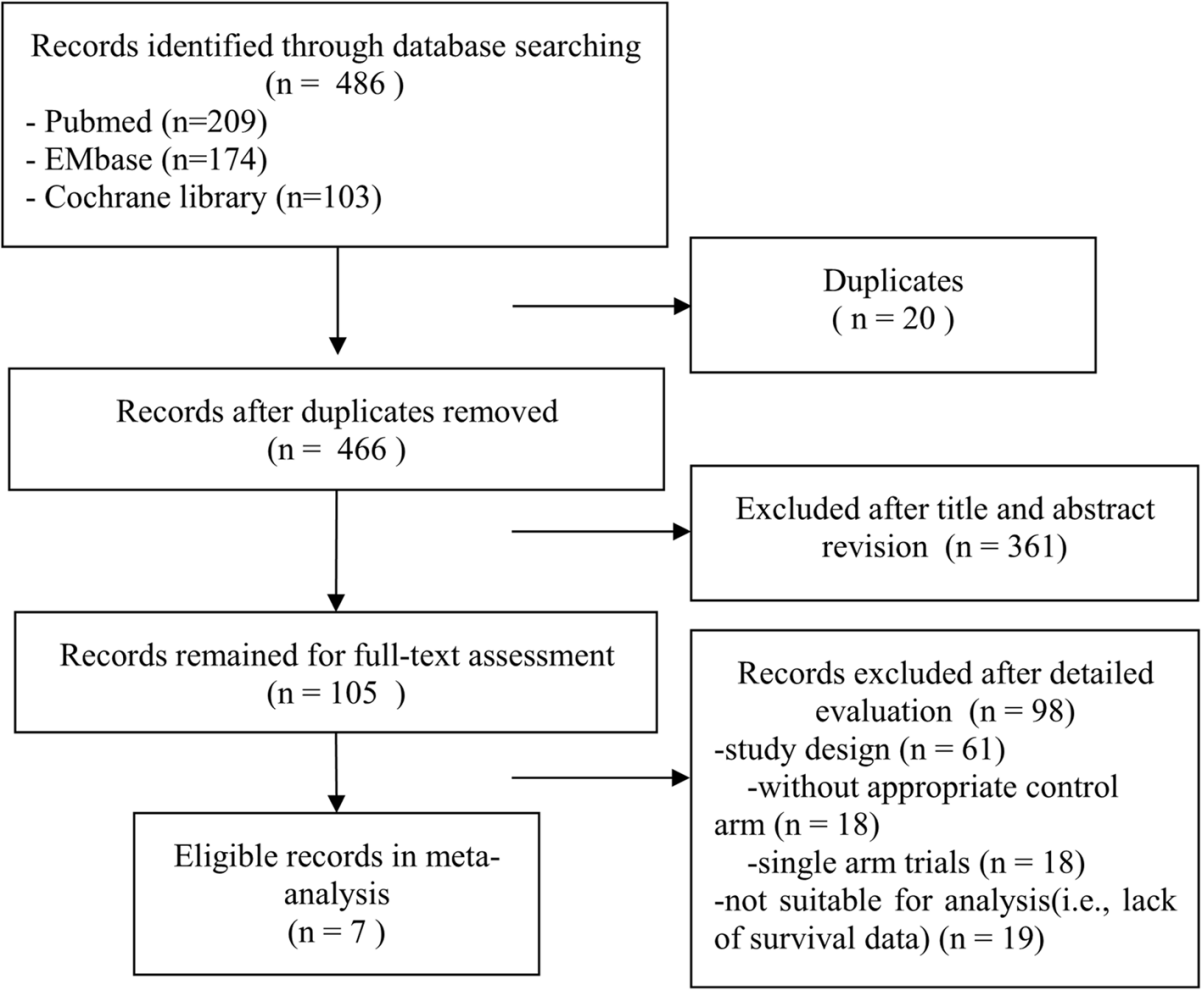
The PRISMA flow diagram

Based on the predefined criteria, we found 486 relevant articles through the search of online databases. Twenty articles were excluded for duplication during the first screening. After title and abstract revision, another 361 articles were excluded. During a full-text assessment, 98 articles were excluded because they were trials without proper control arms, single-arm trials or trials without sufficient data for analyses. Ultimately, seven RCTs (USO 01062, FinXX, GEICAM/2003, CREATE-X, CIBOMA/2004, CBCSG-010 and SYSUCC-001, [16–18, 20, 21, 23, 24], which met the eligibility criteria, were included. The PRISMA flow diagram is shown in Fig. 1.

### Characteristics of enrolled trials

Ultimately, seven RCTs including a total of 3329 early-stage TNBC patients were enrolled in this meta-analysis, with 1640 receiving standard neo−/adjuvant chemo-regimes alone, and the other 1689 patients receiving an additional capecitabine use, respectively. All seven trials have published articles with full text. In 4 trials, capecitabine was “piggybacked” on top of standard chemotherapy regimens, while in the other 3 trials, capecitabine was subsequently administrated after standard chemotherapy regimens.

DFS data could be extracted in six trials except for FinXX, in which RFS was presented. OS data could be extracted in five trials.

The characteristics of the eligible studies are presented in Table 1. The survival data are shown in Table 2.

**Table 1:** Characteristicsof eligible studies

Study	Latest update	Type of trial (Registry number)	Enrollment period	Age Range (year)	Definition of TNBC	Chemo-regimen -Control arm -X arm	TNBC NO. -Control arm -X arm	Capecitabine dosage	TNM stage	Initial situation of patients	Median follow-up
USO 01062	2015	RCT (NCT00089479)	2002.8–2006.2	18–70	ER-, PR-, HER2-	-4 AC-4 T -4 AC-4 TX	−384 − 396	825 mg/m^2^, bid, d1–14, q21d	T_1–3_N_1–2_, or T_1c-4_ N_0_, M_0_	Unselected	5.0y
GEICAM/2003	2015	RCT (NCT00129389)	2004.2–2007.2	18–70	ER-, PR-, HER2-	-4EC-4 T -4ET-4X	− 71 − 95	1250 mg/m^2^, bid, d1–14, q21d	T_1–3_N_1–3_, M_0_	Unselected	6.6y
FinXX	2017	RCT (NCT00114816)	2004.1–2007.5	18–65	ER-, PR-, HER2-	-3 T-3CEF -3TX-3CEX	− 109 − 93	900 mg/m^2^, bid, d1–15, q21d	N_1–3_, or T_2-4_N_0_, M_0_	Unselected	10.3y
CREATE-X	2017	RCT (UMIN000000843)	2007.2–2012.7	20–74	ER-, PR-, HER2-	-standard NACT -standard NACT + 6-8X	−147 − 139	1250 mg/m^2^, bid, d1–14, q21d	T_1-4_N_0–2_, M_0_	Screening from NACT	5.0y
CIBOMA/2004	2019	RCT (NCT00130533)	2006.10–2011.9	20–82	ER-, PR-, HER2-	-standard CT -standard CT + 8X	−428 − 448	1000 mg/m^2^, bid, d1–14, q21d	N_1–3_, or T_1c-4a_N_0_, M_0_	Unselected	7.3y
CBCSG-010	2020	RCT (NCT01642771)	2012.6–2013.12	18–70	ER/PR < 10%+, HER2-	-3 T-3CEF -3TX-3CEX	− 288 − 297	1000 mg/m^2^, bid, d1–14, q21d	N_1–3_, or T_1c-4a_N_0_, M_0_	Unselected	67 months
SYSUCC-001	2020	RCT (NCT01112826)	2010.4–2016.12	24–70	ER-, PR-, HER2-	-standard CT -standard CT + X(1 year)	−213 − 221	650 mg/m^2^, bid, without interruption	T_1b-3_ N_0-3c_, M_0_	Unselected	61 months

*RCT* randomized controlled trials, *TNBC* triple-negative breast cancer, *T* docetaxel, *E* epirubicin, *X* capecitabine, *C* cyclophosphamide, *F* 5-fuorouracil, *A* anthracycline, *No* number, *NACT/CT* neo-adjuvant chemotherapy/chemotherapy

**Table 2:** Survival data of eligible studies

Study	Chemo-regimen -Control arm -X arm	TNBC NO. -Control arm -X arm	HR(95%CI) for Survival data(5-year evaluated) X arm vs Control arm					Subgroup analysis in TNBC patients
			OS	DFS	RFS	LRRFS	DMFS	
USO 01062	-4 AC-4 T -4 AC-4 TX	−384 −396	0.62 (0.41–0.94)	0.81 (0.57–1.15)	NS	NS	NS	NS
GEICAM/2003	-4EC-4 T -4ET-4X	−71 −95	NS	1.19 (0.70–2.04)	NS	NS	NS	All LN positive
FinXX	-3 T-3CEF -3TX-3CEX	−109 −93	NS	NS	0.53 (0.31–0.92)	NS	NS	NS
CREATE-X	-standard NACT -standard NACT + 6-8X	−147 −139	0.52 (0.30–0.90)	0.58 (0.39–0.87)	NS	NS	NS	NS
CIBOMA/2004	-standard CT -standard CT + 8X	−428 −448	0.92 (0.66–1.28)	0.82 (0.63–1.06)	NS	NS	NS	LN status; menopausal status
CBCSG-010	-3 T-3CEF -3TX-3CEX	−288 −297	0.67 (0.37–1.22)	0.66 (0.44–0.99)	0.59 (0.38–0.93)	NS	0.63 (0.39–1.00)	LN status; menopausal status
SYSUCC-001	-standard CT -standard CT + X(1 year)	−213 −221	0.75 (0.47–1.19)	0.64 (0.42–0.95)	NS	0.72 (0.46–1.13)	0.60 (0.38–0.92)	LN status; menopausal status

*NS* not shown

### Efficacy of Capecitabine addition

#### Overall efficiency

**Fig. 2:**
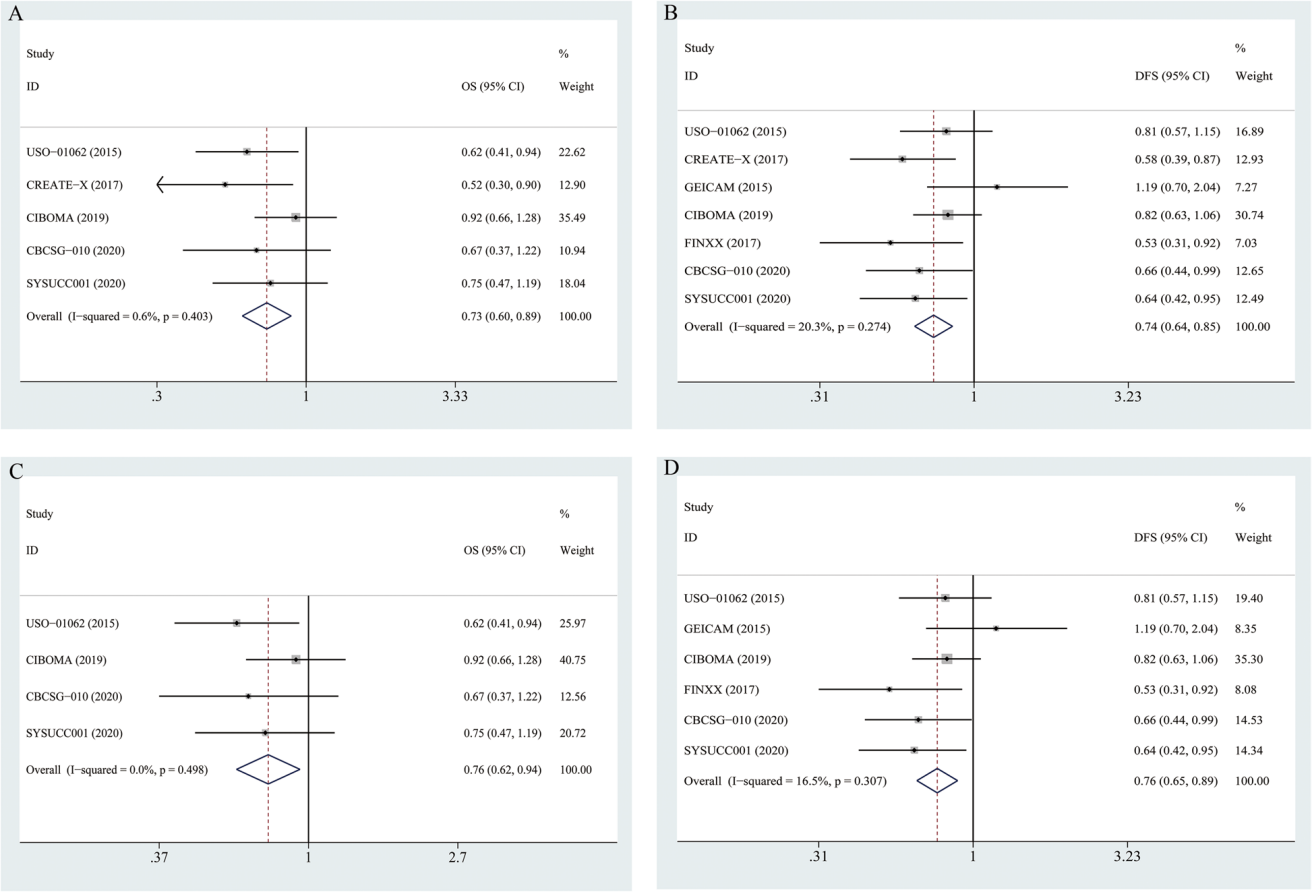
Overall efficiency for additional Capecitabine use in early-staged TNBC. **A** summarized HR for OS in whole TNBC patients, all RCTs included; (**B**) summarized HR for DFS in whole TNBC patients, all RCTs included; (**C**) summarized HR for OS in unselected TNBC patients, with CREATE-X excluded; (**D**) summarized HR for DFS in unselected TNBC patients, with CREATE-X excluded

As shown in Table 2, OS data of TNBC patients were reported in five of the seven RCTs (*n* = 2961 TNBC patients; Capecitabine/X arm: 1501, Control arm: 1460). The separate and summarized HRs and 95%CIs are shown in Fig. 2A. No between-study heterogeneity was noted (*p* = 0.403, I-square = 0.6%). The summarized estimate HR of the capecitabine arm versus the Control arm was 0.73 (95% CI: 0.60–0.89).

All the seven trials provided DFS/RFS data (*n* = 3329; X arm: 1689, Control arm: 1640). Considering that only one trial (FinXX) provided RFS data and that the RFS definition was similar to DFS in the other six trails, RFS in FinXX was calculated as DFS in the pooled effect analysis. The pooled HR was 0.735 (95% CI: 0.637–0.850), as shown in Fig. 2B. A fixed-effects model was used, with no significant between-study heterogeneity (*p* = 0.274, I-square = 20.3%).

The summarized HRs above show that Capecitabine arm has significant improvements in both OS and DFS compared with Control arm. However, as presented in Table 1, the initial situations of TNBC patients recruited across the seven trials were not comparable: one trial (CREATE-X) recruited TNBC patients screened from NACT (with non-pCR after standard NACT), while the other six trials recruited unselected patients. Thus we further repeated the analysis in the trials without CREATE-X.

With the removal of CREATE-X, there were four trials reporting OS data (*n* = 2675; X arm: 1313, Control arm: 1362) and six trials reporting DFS/RFS data (*n* = 3043; X arm: 1550, Control arm: 1493). The summarized HR for OS was 0.76 (0.62–0.94), and that for DFS was 0.76 (0.65–0.89), as shown in Fig. 2C-D.

Altogether, these analyses proved for the first time that the addition of capecitabine to standard chemo-regimens could improve both DFS and OS for early-stage TNBC patients, even for those unselected patients without screening from NACT.

#### Subgroup analysis

**Fig. 3:**
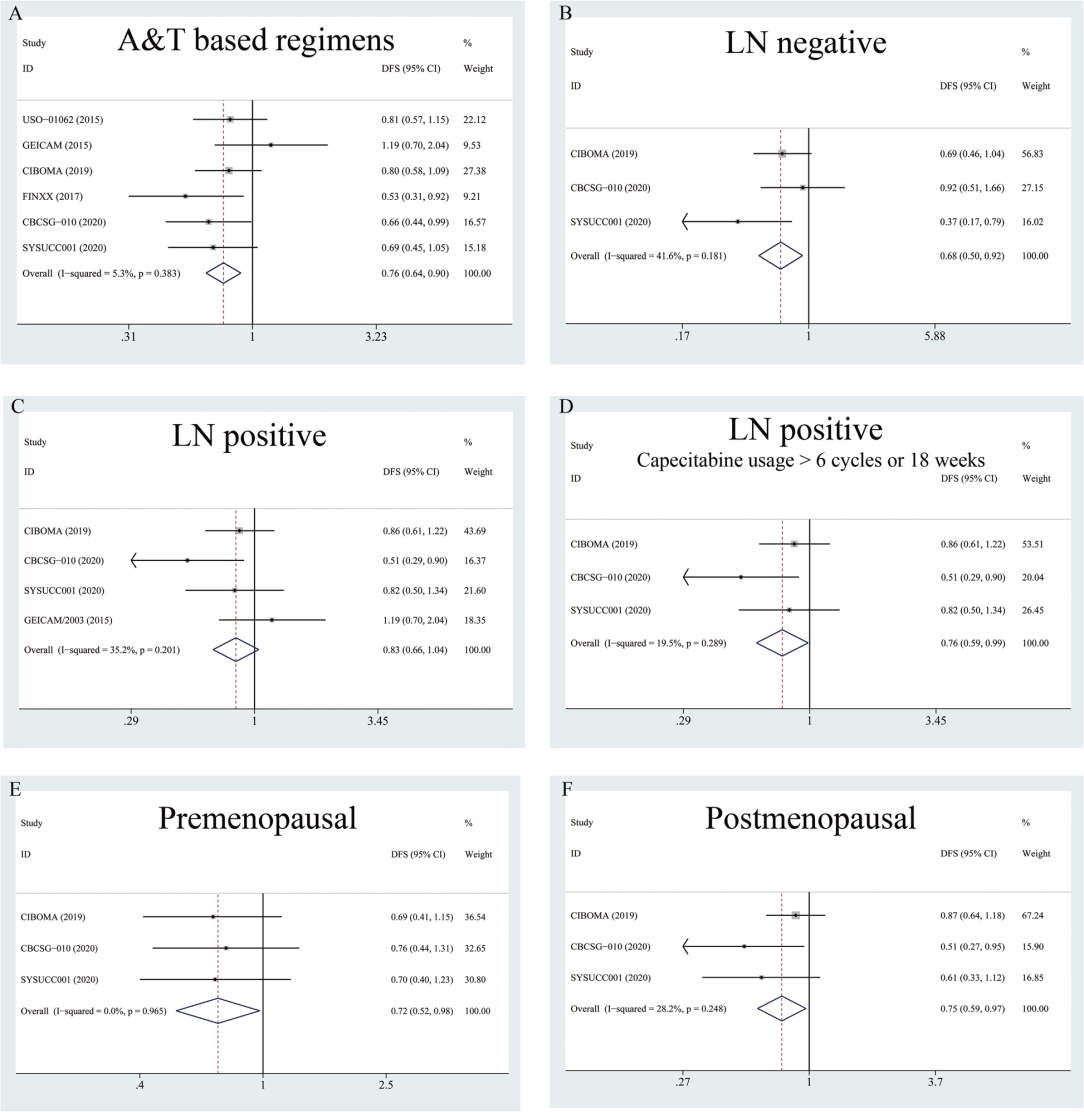
Subgroup analysis for additional Capecitabine use in early-staged TNBC. **A** summarized HR for DFS in unselected TNBC patients receiving A&T based regimens; (**B**) summarized HR for DFS in unselected TNBC patients with LN negative; (**C**) summarized HR for DFS in unselected TNBC patients with LN positive; (**D**) summarized HR for DFS in unselected TNBC patients with LN positive, capecitabine ≥6 cycles or 18 weeks; (**E**) summarized HR for DFS in unselected premenopausal TNBC patients; (**F**) summarized HR for DFS in unselected postmenopausal TNBC patients

Given that some TNBC patients in the CIBOMA/2004 and SYSUCC001 trials received anthracyclines (A) or taxanes (T)-based regimens, we further specifically summarized the HR for DFS in those patients with A&T-based regimens, and the result was accordant (HR = 0.76; 0.64–0.90), see Fig. 3A.

To explore the indications for the addition of capecitabine in unselected early-stage TNBC patients (with the removal of CREATE-X), we further conducted a sub-analysis according to lymph node status and menopausal status.

Three trials (CIBOMA/2004, CBCSG-010 and SYSUCC-001) provided the DFS data in the lymph node-negative (LN negative, LN-) subgroup. As shown in Fig. 3B, for LN- TNBC patients (*n* = 1135; X arm: 575, Control arm:560), a significantly better DFS outcome was indicated in the capecitabine group (HR = 0.673,0.495–0.917). Four trials (GEICAM/2003, CIBOMA/2004, CBCSG-010 and SYSUCC-001) provided the DFS data in the LN-positive (LN+) subgroup (*n* = 913; X arm: 477, Control arm:436). A marginally better result of pooled DFS was found for the capecitabine group (HR = 0.83, 0.66–1.04), see Fig. 3C. Since the GEICAM/2003 trial only add capecitabine for 4 cycles, if we repeated the analysis in the left three trials adding capecitabine≥6 cycles or 18 weeks (*n* = 747; X arm: 382, Control arm:365), the capecitabine group would gain a significantly better DFS (HR = 0.76, 0.59–0.99), see Fig. 3D. Thus, the effect of capecitabine on DFS was definite and consistent across patients with different nodal status in unselected early-stage TNBC patients.

The benefit of capecitabine on DFS was also observed in subgroup analysis of different menopausal status (Fig. 3E-F). Our results proved that both premenopausal (three trials, *n* = 886; X arm: 447, Control arm:439) and postmenopausal (three trials, *n* = 933; X arm: 511, Control arm:482) patients could benefit from adding capecitabine to standard chemotherapy.

#### Toxicity

To evaluate the safety of additional capecitabine in TNBC treatments, we analyzed data from the CBCSG-010, CIBOMA/2004–01 and SYSUCC-001 trials, which only included TNBC patients as the whole cohort. The safety profiles of the capecitabine group and the observation group are listed in Table 3. The grades 3–4 AEs were analyzed. The capecitabine group showed significantly elevated risk Grade 3 or 4 AEs(45.6% vs. 32.8%, OR = 1.17; *p* < 0.001). Hand-foot syndrome (HFS) and neutropenia were the most common AEs. However, severe AEs were rare in both groups. Of note, the CIBOMA/2004–01 trial reported 5 severe AE-caused cases in the capecitabine group with 2 in the observation arm.

**Table 3:** Summarized toxicities data of studies

Events	Grade 3/4, No.(%)		OR (95%CI)	*p* value
	Capecitabine group (*N* = 954)	Control group (*N* = 926)		
HFS	124 (13.0)	0 (0.0)	139.44 (24.43–5555.92)	< 0.001
Neutropenia	137 (14.4)	118 (12.7)	1.13 (0.87–1.48)	0.31
Abdominal pain/diarrhea	18 (1.9)	3 (0.3)	5.92 (1.85–18.88)	< 0.001
Nausea	9 (0.9)	4 (0.4)	2.19 (0.71–6.74)	0.27
Vomiting	17 (1.8)	9 (1.0)	1.85 (0.84–1.08)	0.09
Fatigue	18 (1.9)	2 (0.2)	5.92 (1.85–18.88)	< 0.001
Overall	435 (45.6)	304 (32.8)	1.71 (1.42–2.07)	< 0.001

#### Publication bias

Egger’s test was used to detect the publication bias. There was no significant publication bias identified in the data pooling (results not shown).

## Discussion

Improving the clinical outcomes of early-stage TNBC is still a critical issue [25]. The value of adding capecitabine to standard chemotherapy regimens in non-metastatic TNBC patients has been a hot topic in recent years. There have been several meta-analyses concentrating on this issue [26], of which the latest one was published in 2020 [27]. However, due to limited study quantities and survival data, the previous meta-analysis did not explore the role of capecitabine addition in unselected TNBC patients and failed to identify the target population most likely to benefit from adding capecitabine, especially in those TNBC without screening from NACT.

In this meta-analysis, we included several trials up to date with latest survival data, such as SYSUCC-001, CBCSG-010 and CIBOMA/2004–01, thus making this article more meaningful and convincing with respect to discussing the role of capecitabine in adjuvant treatments of TNBC.

The present research first proved the value of the addition of adjuvant capecitabine for early-stage TNBC patients receiving standard chemotherapy, which is similar to previous studies. Moreover, we explored its value in those unselected TNBC patients (with CREATE-X removal). The summarized HRs for DFS and OS confirmed the value of adding adjuvant capecitabine in unselected TNBC patients receiving standard A&T-based regimens, with the risk decreasing by approximately 1/4.

The subsequent subgroup analysis further ensured the comprehensive benefits of adding capecitabine in unselected TNBC patients, across the LN status and menopausal status. The results even indicated that the addition of adjuvant capecitabine might bring more benefits to LN-negative TNBC patients, decreasing the relapse risk by 33%, compared with a 24% risk decrease in LN-positive group.

**Fig. 4:**
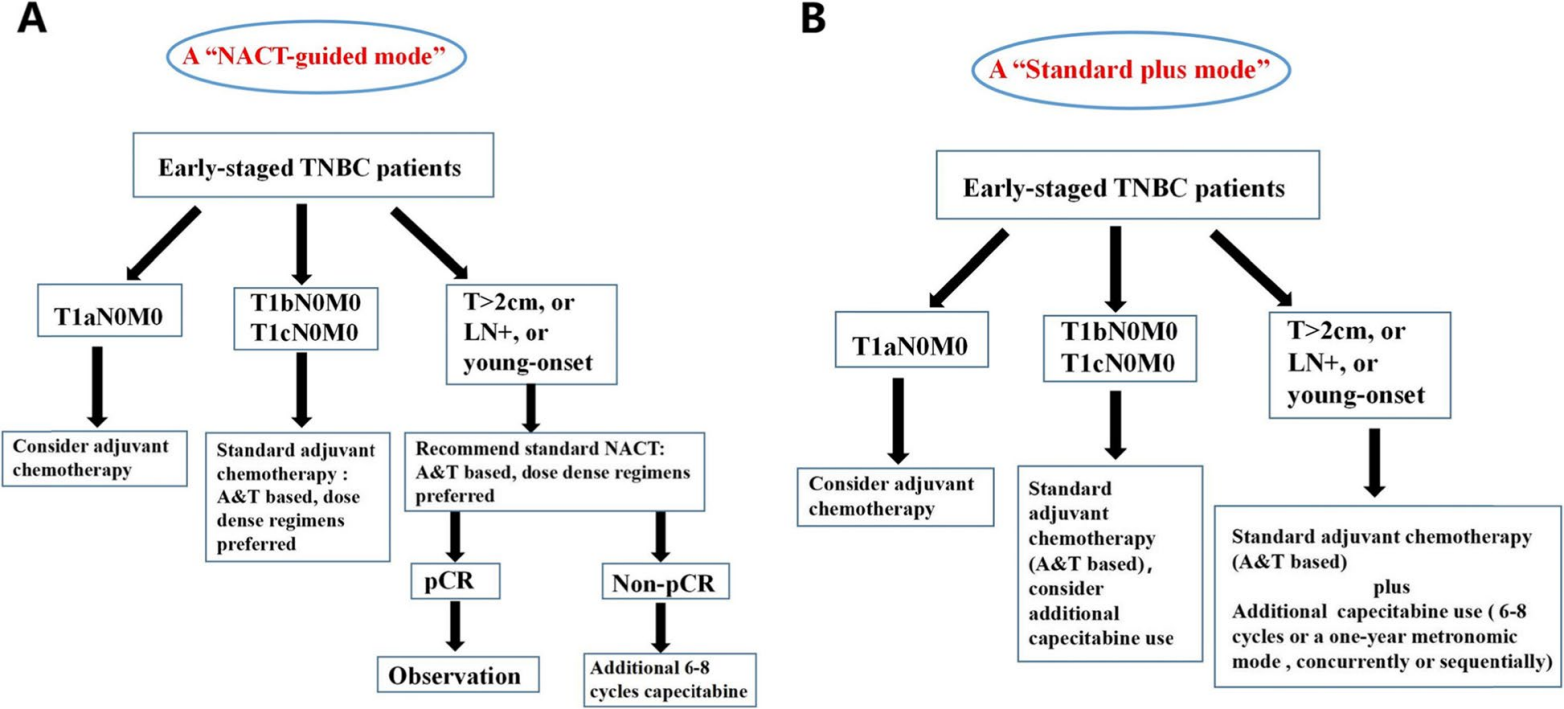
A “NACT-guided mode” or a “Standard plus mode” in early-staged TNBC. **A** In a “NACT-guided mode”, early-staged TNBC patients with risk factors (i.e. T > 2 cm, or LN+, or young-onset (<40y), or high ki67 index) are recommended to receive standard NACT. After screening of NACT, patients with residual invasive tumor are strong candidates for additional 6–8 cycles capecitabine use. **B** In a “Standard plus mode”, standard CT plus capecitabine could be recommended to all early-staged TNBC patients with risk factors, according to the present evidences. For T1b-cN0M0 patients, additional capecitabine could also be considered and discussed with patients

The therapeutic strategies for early-stage TNBC may be profound influenced by these encouraging results. To some extent, the present treatment mode of non-metastatic TNBC is a “NACT-guided mode”. According to the current mainstream guidelines, early-stage TNBC patients with risk factors (i.e., T > 2 cm, or LN+, or young-onset (<40y), or a high ki67 index) are recommended to receive standard NACT. After screening from NACT, patients with residual invasive tumor are strong candidates for additional 6–8 cycles capecitabine use. While in those unselected TNBC patients, indications for capecitabine addition, aside from standard A&T-based regimens, are not clearly defined. However, the present study provides qualified evidence to fill in the blanks. Our results demonstrate that the addition of capecitabine in early-stage TNBC significantly ameliorates prognosis, and the effects are concrete and consistent across LN status and menopausal status. Thus, for non-metastatic TNBC, a new era of “Standard chemotherapy plus capecitabine” is definitely coming (See Fig. 4): we recommend standard A&T based regimens plus additional capecitabine use for early stage TNBC patients with risk factors (T > 2 cm, LN+, young onset); for T1b-cN0M0 patients, additional capecitabine use could be considered, however, the existing data are limited (only in SYSUCC-001 with a very small sample size).

In summary, the “NACT-guided mode” is still the preferred therapeutic algorithm for early-stage TNBC with risk factors. However, in practical work, when we encounter TNBC patients who undergo breast surgery first, the “Standard chemotherapy plus capecitabine” mode could play a role. Thus, this recommendation is not a replacement but a supplement for the present therapeutic algorithm. These two modes could work together to make eTNBC treatments more comprehensive.

Regarding the usage of capecitabine in unselected early stage TNBC patients, we think there are no consensuses reached thus far, since most existing trials (as shown in Fig. 2C, a 6–8 cycles mode in the CIBOMA 2019 and CBCSG-010 trials, while a one-year metronomic mode in SYSUCC-001) are all negative for an OS benefit. Thus we recommend both based on the existing trials. Both of these two modes show tolerable toxicities.

Our study has several limitations. First, the RCTs included in the present study used different dosages and treatment duration of capecitabine and which one should be the optimal treatment plan remains unclear. Second, the present study did not discuss how to treat those TNBC patients who achieve pCR after NACT. In our opinion, pCR predicts a favourable but unsatisfactory prognosis for TNBC, with a 5-year DFS rate of approximately 85% [9]. On the other hand, TNBC patients who tend to receive NACT always have risk factors, then why do not we consider addition of capecitabine, since our data support the application of capecitabine in all stage TNBC patients, irrespective of nodal status and menopausal status. Third, TNBC has been demonstrated to be a highly heterogeneous disease with various intrinsic characteristics in recent years [28–30]. However, information on these important genomic or metabolic characteristics was deficient in the eligible trials. Thus which specific subtype of TNBC would benefit the most from adding capecitabine remains unclear, too. Finally, detailed risk stratification and efforts to identify patients at a higher risk are needed. Highly sensitive diagnosis tests, such as circulating tumor cells and circulating tumor DNA technology might be promising in this regard.

## Conclusions

The present meta-analysis of RCTs demonstrates for the first time that adding capecitabine to standard chemo-regimens could improve both DFS and OS in unselected eTNBC patients, and this benefit remains consistent regardless of nodal status and menopausal status, which may lead eTNBC therapy into a new era.

## Data Availability

The data used to support the findings of this study are included within the article.

## Abbreviations

TNBC: Triple negative breast cancer
eTNBC: Early-staged TNBC
RCT: Randomized controlled trial
NACT: Neoadjuvant chemotherapy
HR: Hazard ratios
95%CI: 95% confidence interval
ER: Estrogen receptor
PR: Progesterone receptor
A/E: Anthracyclines
T: Taxanes
C: Cyclophosphamide
Cb: Carboplatin
X: Capecitabine
5-Fu/F: Fluorouracil
PRISMA: Preferred Reporting Items for Systematic Reviews and Meta-analyses
PROSPERO: International Prospective Register of Systematic Reviews
ASCO: American Society of Clinical Oncology
ESMO: The European Society for Medical Oncology
SABCS: San Antonio Breast Cancer Symposium
DFS: Disease-free survival
OS: Overall survival
RFS: Relapse free survival
LRRFS: Local-regional recurrence free survival
DMFS: Distant metastasis-free survival
